# Exploring the Potential of the Microbiome as a Marker of the Geographic Origin of Fresh Seafood

**DOI:** 10.3389/fmicb.2020.00696

**Published:** 2020-04-17

**Authors:** Xiaoji Liu, Januana S. Teixeira, Saurabh Ner, Kassandra V. Ma, Nicholas Petronella, Swapan Banerjee, Jennifer Ronholm

**Affiliations:** ^1^Faculty of Agricultural and Environmental Sciences, McGill University, Sainte-Anne-de-Bellevue, QC, Canada; ^2^Health Canada, Ottawa, ON, Canada

**Keywords:** microbiome, seafood, adulteration and substitution, mothur, food fraud prevention

## Abstract

Geographic food fraud – misrepresenting the geographic origin of a food item, is very difficult to detect, and therefore this type of fraud tends to go undetected. This potentially negatively impacts the health of Canadians and economic success of our seafood industry. Surveillance studies have shown that up to a significant portion of commercially sold seafood items in Canada are mislabeled or otherwise misrepresented in some way. The current study aimed to determine if the microbiome of fresh shellfish could be used as an accurate marker of harvest location. Total DNA was extracted from the homogenate of 25 batches of fresh soft-shell clams (*Mya arenaria*) harvested in 2015 and 2018 from two locations on the East Coast of Canada and the microbiome of each homogenate was characterized using 16S rRNA targeted amplicon sequencing. Clams harvested from Nova Scotia in both years had a higher abundance of *Proteobacteria* and *Acidobacteria* (*p* < 0.05), but a lower abundance of *Actinobacteria* (*p* < 0.05) than those from Quebec. Alpha-diversity also differed significantly between sites. Samples harvested from Nova Scotia had greater diversity (*p* < 0.0001) than those from Quebec. Beta-diversity analysis showed that the microbial community composition was significantly different between the samples from Nova Scotia and Quebec and indicated that 16S rRNA targeted amplicon sequencing might be an effective tool for elucidating the geographic origin of unprocessed shellfish. To evaluate if the microbiome of shellfish experiences a loss of microbial diversity during processing and storage – which would limit the ability of this technique to link retail samples to geographic origin, 10 batches of retail clams purchased from grocery stores were also examined. Microbial diversity and species richness was significantly lower in retail clams, and heavily dominated by *Proteobacteria*, a typical spoilage organism for fresh seafood, this may make determining the geographic origin of seafood items more difficult in retail clams than in freshly harvested clams.

## Introduction

Globally, seafood is one of the most common food categories associated with fraud (Pardo et al., 2016; Van Ruth et al., 2018). Widespread fraud undermines the credibility and profitability of this industry which currently generates US$150 billion in economic activity annually (FAO, 2018). High rates of seafood fraud are the cumulative result of several factors: seafood is the most highly traded food commodity, it has a long and complex supply chain, and most of the supply chain is not readily transparent (Kittinger et al., 2017). International laws entitle consumers to know the identity of the species, the geographical origin, and the production method (caught or farmed) for all categories of seafood (Chatterjee et al., 2019). Seafood fraud includes any type of intentional mislabeling that misleads the consumer about any of these parameters. The consequences of producing mislabeled seafood are broad but can include human health risks, economic losses, and undermining sustainability efforts. For example, escolar mislabelled as albacore tuna can cause diarrhea and vomiting due to the laxative effects of the indigestible oil found in the former (Escolar and Adverse Effects, Health Canada, 2008). In addition, human health is also placed at risk by the presence of pathogens or other banned substances, such as antibiotics, in fraudulent seafood (Feldhusen, 2000). Economically Atlantic salmon mislabeled as sockeye salmon, which is preferred by customers due to its delicate flavor and texture, results in consumers paying more for a less valuable product (Oceana Canada, 2018).

It is difficult to estimate the true global burden of seafood fraud; however, the percentage of mislabeled seafood is potentially very high. A recent meta-analysis, which considered data from the United States, Italy, Spain, Brazil, and the United Kingdom, found that at the product-level the mislabeling rate is approximately 8% (Luque and Donlan, 2019). However, this meta-analysis also revealed that some species had particularly high mislabeling rates – such as the Northern Red Snapper that was mislabeled 74% of the time and was commonly being substituted with aquaculture species (Luque and Donlan, 2019). A slightly higher rate of 12.9% was detected in a 3-year study carried out to detect species mislabeling in the Greek Market (Minoudi et al., 2020), and a slightly lower rate was detected in Spain which found a mislabeling rate of 6.2% (Helgoe et al., 2020). In Canada, a recent survey sampled 12 target marine species (*n* = 203) from retailers, importers, and processing plants in Ontario and found that 32% were mislabeled at some point during production (Shehata et al., 2019).

The current method for detection of species substitution is DNA barcoding. This technique is relatively straightforward and based on sequencing the partial cytochrome c oxidase subunit I (*COI*) gene, which allows accurate species identification (Hebert et al., 2003; Staffen et al., 2017; Shehata et al., 2019). Detection of geographic fraud is more difficult – and thus fewer studies to determine the prevalence of this type of food fraud have been conducted. Isotope Ratio Mass Spectrometry (IRMS) analysis is being investigated as a possible technique to detect geographic fraud. This technique profiles the isotopes of elements associated with food products, in particular the ratio of ^18^O/^16^O, from different geographic regions (Kelly, 2003; Black et al., 2016). The strength of this technique is that it is universal and can be used for any food product that contains water; however, the key weaknesses of this technique are that great effort is required initially to build a database of the isotopes associated with various geographic localities, and the materials required for performing IRMS are very expensive. It is therefore difficult to use this technique for routine surveillance (Rossier et al., 2014). IRMS has been widely successfully used for geographic delineation of several processed high-value processed food products such as honey (Zhou et al., 2018), fruit juice (Dasenaki and Thomaidis, 2019), and wine (Christoph et al., 2015); and although IRMS is effective for a broad range of products, for raw unprocessed food products – particularly those being harvested from the environment, geographic origin may be more easily elucidated by using the composition of the microbiome as a marker.

The microbiome of shellfish such as mussels, oysters, and clams varies based on geographic location, season, temperature, salinity, and other environmental conditions (King et al., 2012; Chauhan et al., 2014; Lokmer and Wegner, 2015; Pierce et al., 2016; Pierce and Ward, 2018; Stevick et al., 2019). As filter feeders, the microbiome of shellfish is thought to be heavily influenced based on which bacteria are consumed from the environment (Trabal et al., 2012); although, the microbiome of shellfish can be significantly different from surrounding seawater, indicating that host-microbiome interactions are also important (Vezzulli et al., 2017). In this study, we have hypothesized that the geographic harvest site impacts the composition of the shellfish microbiome to such an extent that it can be used reliably to differentiate shellfish harvested from different locations. As a proof of principle, fresh soft-shell clams were harvested from two sites on the Canadian East Coast, one in Quebec and one in Nova Scotia separated by approximately 800 km. The bacterial community was characterized by 16S rRNA targeted amplicon sequencing to identify the signature taxa associated with a specific harvest site. Samples harvested over a 3-year span could reliability be differentiated by harvest site based on the composition of the microbiome. However, the microbial diversity was drastically reduced in retail samples, which had been processed and subjected to storage – indicating that this may not be a straightforward technique in standard commercial settings.

## Materials and Methods

### Sampling and Data Collection

Fresh clams: 25 batches of 10–15 soft-shell clams (*Mya arenaria*) were harvested from two different sites on the east coast of Canada, Gaspé (Sainte-Jean River) in Quebec and Smith’s Cove in Nova Scotia, during the clam harvesting season (May to October) of 2015 and 2018. Upon harvest, all samples were immediately stored on ice and shipped directly to the Bureau of Microbial Hazards at Health Canada (Ottawa, ON), where they were then homogenized and frozen until DNA extraction could be performed.

Retail clams: 10 batches of 10–15 fresh, minimally processed clams, stored and sold on ice, were purchased from various grocery stores at different time-points throughout November 2014 to February 2016. Among the ten batches, nine were confirmed to have been harvested on the West coast, and one from the East coast – but more detailed information was not available. No information was available on whether those samples had gone through depuration or how long the samples had been stored at the time of purchase. Upon purchase, retail clams were transported on ice to the laboratory and homogenized. The homogenate was kept frozen until DNA extraction could be performed.

### Sample Preparation and DNA Extraction

Clams were carefully shucked under aseptic conditions. The entire batch was placed into a sterile blender, homogenized, frozen and stored until further extraction. The blenders were autoclaved, washed, and then re-autoclaved and stored under sterile conditions between each use. Total DNA was extracted by using DNeasy Food Extraction Kit (QIAGEN, Germany) following the manufacturer’s instructions. The concentration and purity of extracted DNA were assessed using Nanodrop ND-1000 (Thermo Scientific, MA). All samples yielded total DNA concentrations exceeding 5 ng/μl.

### Library Preparation, PCR Amplification and Sequencing

Paired-end sequencing was used to analyze the bacterial community composition of each sample using the V4 region of the 16S rRNA gene as a proxy for total bacterial diversity. Briefly, the V4 region of the 16S rRNA gene was amplified from total DNA in the sample using a set of custom primers, F548 and R806, that included Illumina indexing which was later used in demultiplexing as described in Kozich et al. (2013). Each sample was amplified via PCR with 25 cycles of 95°C for 20 s, 55°C for 15 s, and 72°C for 1 min, followed by a final extension at 72°C for 7 min and then holding the samples at 4°C. The PCR products were purified using AMPure XP beads (Beckman Coulter) according to the manufacturer’s instructions and quantified using the Quant-iT^TM^ dsDNA High-Sensitivity Assay Kit (Thermo Fisher Scientific). Any amplicons that were found to have a concentration of less than 1.5 ng/μL were re-amplified from the total extracted DNA. After quantification, the amplicons were diluted using DNA/RNA free water to a final concentration of 1.5 ng/μL and a sequencing library was prepared by combining each sample. The 16S rRNA amplicons were sequenced for 250 bp in both forward and reverse directions.

### Data Analyses

The sequence reads were processed in Mothur (v.1.42.3; Schloss et al., 2009) on the Niagara supercomputer at the SciNet HPC Consortium (Loken et al., 2010; Ponce et al., 2019). Operational taxonomic unit (OTU) picking was performed against SILVA ribosomal RNA database (132 release; Quast et al., 2013; Chen et al., 2018). Data interpretation, statistical analysis, and visualization were performed in MicrobiomeAnalyst (Dhariwal et al., 2017), including rarefaction to the minimum library size without scaling of data (Liu et al., 2017; Kaczmarek et al., 2019). Calculation of the alpha diversity scores, abundance-based Bray-Curtis dissimilarity (beta diversity), ordination via Non-Metric Multidimensional Scaling (NMDS), and linear discriminant analysis effect size (LEfSe) analysis (Segata et al., 2011) were each preformed using MicrobiomeAnalyst (Dhariwal et al., 2017).

### Statistical Analyses

Kruskal-Wallis tests were performed (Liu et al., 2017) comparing the alpha diversity scores between samples from Quebec and Nova Scotia. Multiple *t*-tests were performed comparing the abundance of each taxa in samples harvested from two sites in 2015/2018 (Graphpad Prism 8.2.1). The analysis of similarities (ANOSIM) and permutational multivariate analysis of variance (PERMANOVA) were performed for the beta diversity analyses.

## Results

### The Microbiome Composition of Fresh Clams Is Distinct Between Sites Over Time

To assess if the composition of the microbiome was significantly different between clams from different geographic origins, 25 batches of fresh clams were harvested from either Quebec or Nova scotia. A total of 1,141,528 sequence reads passed filter with an average count of 45,661 per sample. A total of 2,994 OTUs with counts ≥2 were generated.

**FIGURE 1:**
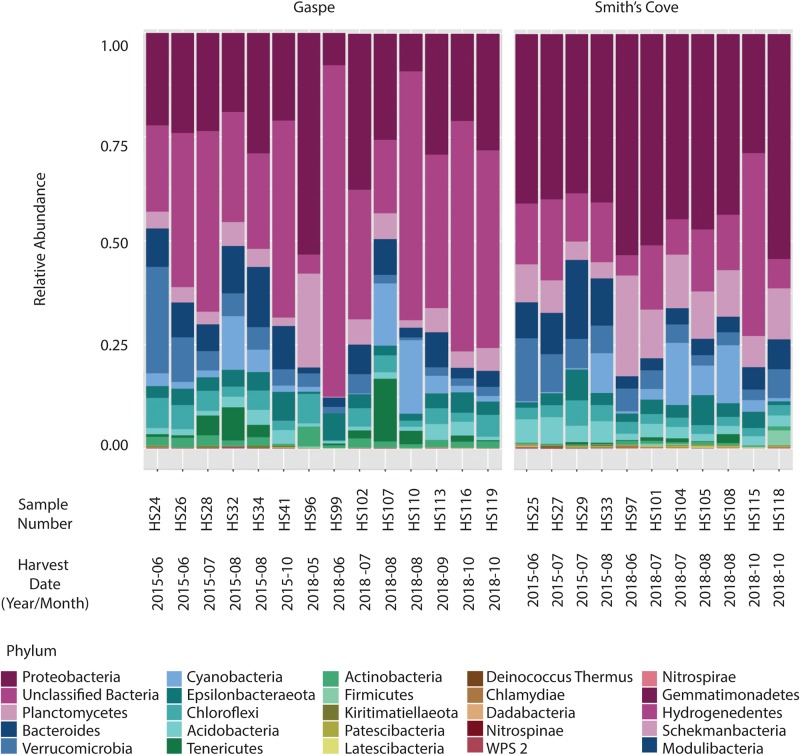
Relative abundance at the phylum level of microbiome associated with fresh clams harvested from Gaspe and Smith’s Cove.

**FIGURE 2:**
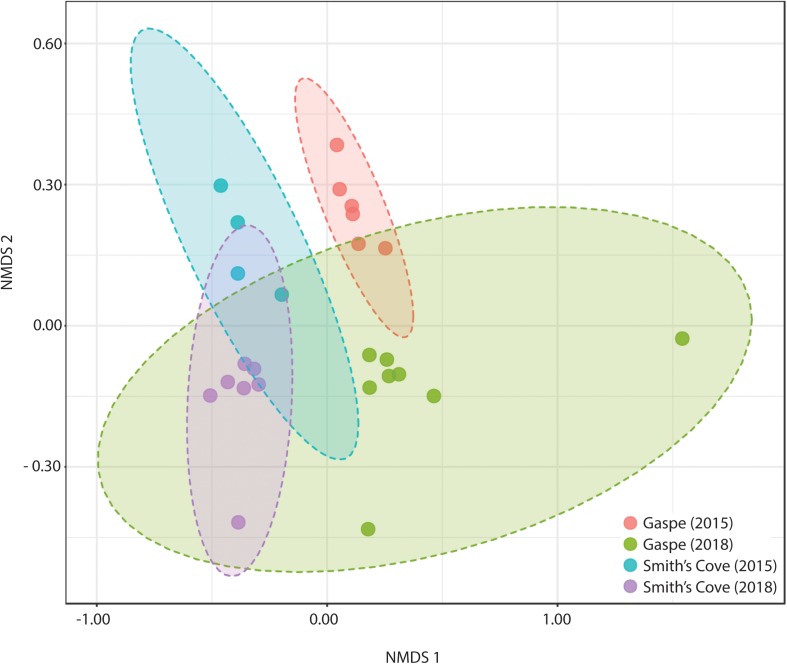
Non-metric Multidimensional Scaling (NMDS) ordination of beta-diversity analyses at the OTU level of fresh clam microbiome communities grouped by harvest site and year: ANOSIM R: 0.631; PERMANOVA *F*-value: 5.724; *R*-squared: 0.450; *p* < 0.001. NMDS ordination stress = 0.099.

**FIGURE 3:**
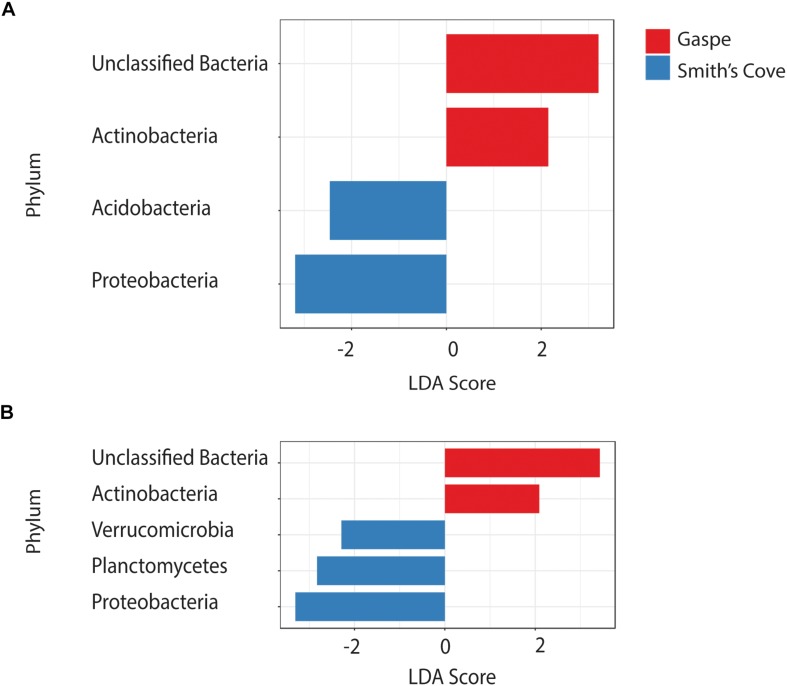
LEfSe analysis showing the discriminatory phyla in the microbiome associated with clams harvested from Quebec and Nova Scotia in **(A)** 2015 and **(B)** 2018. FDR adjusted *p*-value cut off = 0.1.

For fresh clam samples harvested in both 2015 and 2018, *Proteobacteria* was the most abundant classified phylum, followed by *Bacteroidetes* and *Planctomycetes* (Figure 1). The composition of the microbiome was significantly different between harvest locations. Both the Shannon and Simpson indices of alpha-diversity were significantly higher from samples collected in Nova Scotia (4.73 and 0.97, respectively) than those from Quebec (3.79 and 0.88, respectively; *p* < 0.0001, Kruskal-Wallis statistic: 10; Table 1). No significant difference in Chao1 scores were found between samples collected from each site (*p* = 0.075, Kruskal-Wallis statistic: 110). For beta-diversity, ANOSIM and PERMANOVA analysis of Bray-Curtis dissimilarities based on location identified significant dissimilarities between the two sites (ANOSIM *R* = 0.688, *p* < 0.001; PERMANOVA *F* = 9.341, *p* < 0.001; R-squared: 0.289). NMDS ordination (ordination stress = 0.096) provides an excellent visual representation of this distinctive clustering pattern of the microbiome communities based on geographic locations in reduced dimensions (Figure 2). Such dissimilarities between the microbiome communities were seen between samples harvested in both 2015 and 2018. LEfSe analysis showed that *Proteobacteria* was the phylum that contributed most to the distinction in the microbiome community between clams harvested from different sites in both 2015 and 2018 (Figures 3A,B). More specifically, *Desulfobulbaceae* and *Halieaceae* were the two families from *Proteobacteria* phylum that contributed to these differences (LDAScore < −2, FDR adjusted *p*-value cut off = 0.1).

**TABLE 1:** Alpha-diversity indices of microbiome at the operational taxonomic unit (OTU) level associated with fresh clams [harvested from Quebec (*n* = 14) and Nova Scotia (*n* = 11)] and retail samples (*n* = 10).

Harvest site/Category	Chao1	Shannon	Simpson
Fresh (Quebec)	532.90^a^ ± 30.69	3.79^a^ ± 0.21	0.88^a^ ± 0.02
Fresh (Nova Scotia)	482.58^a^ ± 26.08	4.73^b^ ± 0.13	0.97^b^ ± 0.01
Retail	45.12^b^ ± 15.96	1.23^c^ ± 0.18	0.52^c^ ± 0.07

*Results are Mean ± SE. Letters represent statistical significance (*P* < 0.05) in each column.*

### Alpha-Diversity of the Microbiome Was Significantly Lower in Retail Clams

To further explore how the microbiome differs between retail and freshly harvested clams, and evaluate if the microbiome would be a good marker of origin in retail clams we evaluated alpha- and beta-diversity in 10 batches of retail clams purchased from various grocery stores. A total of 1,408,948 reads with an average count of 140,894 per batch of samples were obtained. A total of 149 OTUs with counts ≥ 2 were generated.

**FIGURE 4:**
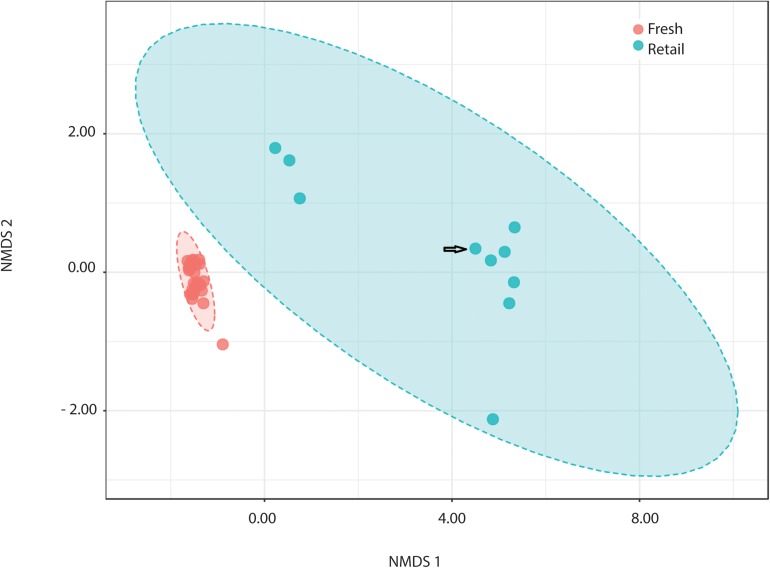
Comparison of retail clams with fresh harvest by NMDS ordination of beta-diversity analysis at the OTU level (ANOSIM R: 0.949; PERMANOVA *p* < 0.001). NMDS ordination stress = 0.045. Arrow indicates the batch of retail clams from the Atlantic. The rest nine batches of retail samples were from the Pacific.

All measures alpha-diversity, including Chao1 (*p* < 0.0001, Kruskal-Wallis statistic: 250), Shannon index (*p* < 0.0001, Kruskal-Wallis statistic: 250), and Simpson index (*p* < 0.0001, Kruskal-Wallis statistic: 246) showed significantly less species richness and overall diversity in retail samples than in fresh samples (Table 1). *Proteobacteria* dominated the average of retail sample, which contributed to the clear separation between the microbial communities in fresh and retail samples (Figure 4). Specifically, the genus *Vibrio* was found at a rate of 20.09% of the microbial population retail samples while only 0.03% in fresh samples. The *Vibrio* genus was the major factor that contributed to the differences between fresh and retail samples (LDAScore < −2, FDR adjusted *p*-value cut off = 0.1).

## Discussion

Our study assessed the potential of using the microbiome of raw clams as a marker of the geographic location of harvest. We found that the composition of the microbiome from clams harvested from two different sites 800 km apart on the Canadian East Coast was distinctive, and that this difference was stable over at least 3-years. Previous studies have shown that 16S rRNA targeted amplicon sequencing was able to identify the specific pond from which cultured seabass originated (Pimentel et al., 2017), and a similar approach, combined with machine-learning was also able to trace the geographic origin of Manila clams (Milan et al., 2019) indicating that this technology may have value in the tracking and identification of geographic food fraud.

A major hurdle to using the microbiome as a maker of the geographic location of various seafood commodities will be developing a database of the expected microbiome from various locations to compare samples to. Indeed, this is one of the hurdles associated with techniques that provide comparable information such as IRMS (Rossier et al., 2014). Existing databases such as Barcode of Life provide reference points for species identification but did take significant effort to build (Ratnasingham and Hebert, 2007). However, our pilot analysis has indicated the stability of the microbiome over time, and therefore these databases would not seem to require annual up-dating.

The microbiome of wild shellfish is influenced by a variety of environmental conditions (King et al., 2012; Chauhan et al., 2014; Lokmer and Wegner, 2015; Pierce et al., 2016; Pierce and Ward, 2018; Stevick et al., 2019), and linking specific environmental conditions to microbiome composition may be interesting. For example, we determined that the *Desulfobulbaceae* and *Halieaceae* families contributed to the differences between clams harvested from different geographic sites. *Desulfobulbaceae* reduces sulfates and assimilate acetate (Dyksma et al., 2018), and potentially recycle iron (Reyes et al., 2016) in marine sediments. Strains from the *Halieaceae* family have been shown to assimilate alkene in surface seawater (Suzuki et al., 2019). It would be interesting for future research to examine whether the inorganic and organic matters in the sediment and surface water of Quebec and Nova Scotia impact the abundance of *Desulfobulbaceae* and *Halieaceae*.

Another possible drawback of using the microbiome as a marker of geographic origin of seafood may be that the composition may change drastically during depuration, processing, and retail storage. The clams used in this study were fresh, never frozen, and minimally processed. However, after harvest, in Canada, most species of bivalve mollusks, including the clams examined in this study, are subjected to a depuration process that is regulated by the Canadian Food Inspection Agency (Government of Canada, 2012). As they grow shellfish concentrate contaminants from the water column, including chemical contaminants as well as bacterial and viral pathogens. Depuration is a process in which shellfish are held in tanks of clean seawater prior to retail, and this results in expulsion of the intestinal contents and improves the safety of the final product (Lee et al., 2008). After depuration, fresh clams (in the shell) typically have a shelf life of 2,3 days (BC Centre for Disease Control, 2019). However, Canadian regulations allow for variation in the depuration process, such as allowing for up to 21 days to pass between harvest and the depuration process if clams are stored in an appropriate wet-storage facility, which may introduce even more variability into the composition of the microbiome.

During retail and consumer storage, clams are often spoiled by *Vibrio*, *Pseudomonas*, *Aeromonas*, and coliforms (Martínez et al., 2009). We do not have information on the type(s) of processing that the retail samples had gone through or how long the samples were stored prior to purchase. To test the hypothesis if processing and storage reduced the diversity of the microbiome – and therefore may have affected the usefulness of this analysis as a geographic food fraud detection tool, we assessed the microbiome of retail clams. We observed significant *reductions* in alpha-diversity between fresh clams and retail clams. A clear indication of this was that 2,994 OTUs were identified in fresh clams, while at a much greater sequencing depth only 149 unique OTUs were found in retail clams. The processing and storage conditions likely favored the growth of *Proteobacteria* resulting in a loss of microbial diversity which will possibly hinder the ability of 16S rRNA targeted amplicon sequencing to resolve the geographic origin of processed shellfish. This finding was consistent with previous findings that showed the microbiomes of shellfish become more similar as they spoil (Madigan et al., 2014). Other various processing and preservation strategies, including ozone treatment, organic acids, phage treatment, high pressure processing, and irradiation will also likely impact the microbiome of seafood (Ronholm et al., 2016). For example, ozonation affected populations of *Pseudomonas* spp. and *Enterobacteriaceae* (Manousaridis et al., 2005). Citric acid or lactic acid resulted in a 5-log reduction of *Vibrio vulnificus* inoculated in fresh shucked oysters (Mahmoud, 2014). The differences in habitat between the marine environment and those experienced at retail outlets, particularly the psychrophilic bias at retail, will also change the microbiome in the products. Thus, depuration, spoilage, and preservation techniques will likely impact the sensitivity for detecting geographic fraud using 16S rRNA targeted amplicon sequencing in retail samples.

In the future, it would be interesting to apply finer bioinformatic analyses on more seafood samples, such as machine learning analysis, to predict the provenance (such as the Random Forest classifier implemented in the sample-classifier QIIME2 plugin to predict a categorical sample metadata category; Chen et al., 2018). Further, it is important to extend the microbiome analysis to the entire food chain starting from sea to market to better understand where the change in microbiome community happens along the seafood processing line and the corresponding food safety consequences.

## Data Availability Statement

Raw sequence reads for each batch have been deposited to Sequence Read Archive (SRA) under the BioProject PRJNA575196 (accession nos. SRX6937767 to SRX6937801).

## Author Contributions

JR oversaw the project and performed sample handling and preparation for sequencing. XL performed the data and statistical analyses, and wrote the manuscript. JT, SN, KM, and NP supported in proofreading and writing. SB provided funding support and access to research facilities.

## Conflict of Interest

The authors declare that the research was conducted in the absence of any commercial or financial relationships that could be construed as a potential conflict of interest.
